# Multiple Sclerosis, Relapses, and the Mechanism of Action of Adrenocorticotropic Hormone

**DOI:** 10.3389/fneur.2013.00021

**Published:** 2013-03-08

**Authors:** Amy Perrin Ross, Aliza Ben-Zacharia, Colleen Harris, Jennifer Smrtka

**Affiliations:** ^1^Department of Neurosciences, Loyola University ChicagoChicago, IL, USA; ^2^Mount Sinai Medical CenterNew York, NY, USA; ^3^Multiple Sclerosis Clinic, Department of Clinical Neurosciences, University of CalgaryCalgary, AB, Canada; ^4^Fort Lauderdale MS CenterPompano Beach, FL, USA

**Keywords:** multiple sclerosis, melanocortins, adrenocorticotropic hormone, anti-inflammatory, immune modulation, MS relapses, corticosteroids

## Abstract

Relapses in multiple sclerosis (MS) are disruptive and frequently disabling for patients, and their treatment is often a challenge to clinicians. Despite progress in the understanding of the pathophysiology of MS and development of new treatments for long-term management of MS, options for treating relapses have not changed substantially over the past few decades. Corticosteroids, a component of the hypothalamic-pituitary-adrenal axis that modulate immune responses and reduce inflammation, are currently the mainstay of relapse treatment. Adrenocorticotropic hormone (ACTH) gel is another treatment option. Although it has long been assumed that the efficacy of ACTH in treating relapses depends on the peptide’s ability to increase endogenous corticosteroid production, evidence from research on the melanocortin system suggests that steroidogenesis may only partly account for ACTH influences. Indeed, the melanocortin peptides [ACTH and α-, β-, γ-melanocyte-stimulating hormones (MSH)] and their receptors (Melanocortin receptors, MCRs) exert multiple actions, including modulation of inflammatory and immune mediator production. MCRs are widely distributed within the central nervous system and in peripheral tissues including immune cells (e.g., macrophages). This suggests that the mechanism of action of ACTH includes not only steroid-mediated indirect effects, but also direct anti-inflammatory and immune-modulating actions via the melanocortin system. An increased understanding of the role of the melanocortin system, particularly ACTH, in the immune and inflammatory processes underlying relapses may help to improve relapse management.

## Introduction

Relapsing-remitting multiple sclerosis (RRMS) is the most common subtype of MS, accounting for over 80% of initial MS diagnoses. Patients experience relapses (also called attacks or exacerbations or flare-ups), separated by periods of full or partial recovery (Lublin and Reingold, 1996; Confavreux et al., 2000). Relapses may lead to the accumulation of disability, and patients usually do not return to their baseline (Lublin et al., 2003). Although relapses are common and represent a defining feature of MS, there remain gaps in our understanding of their clinical management.

Over the last decade, the mainstay of treatment for relapses has been high-dose corticosteroids (National Clinical Advisory Board of the National Multiple Sclerosis Society, 2008). Corticosteroid treatment shortens the time to recovery from relapses (Filippini et al., 2000; Burton et al., 2009), presumably due at least in part to their anti-inflammatory effects. However, corticosteroids are not the sole option for the treatment of relapses. Adrenocorticotropic hormone (ACTH) gel (H.P. Acthar^®^ Gel, 2011), a long-acting formulation of the full sequence ACTH_(1–39)_ that includes other pro-opiomelanocortin (POMC) peptides, is considered an alternative to steroids in treatment of optic neuritis and acute exacerbations of MS, as well as other conditions (H.P. Acthar^®^ Gel, 2011). Before recognition of melanocortin receptors (MCR), ACTH effects were believed to depend on induction of endogenous corticosteroid production (National Clinical Advisory Board of the National Multiple Sclerosis Society, 2008; Sibley, 2009). However, recent evidence suggests that ACTH has additional actions independent of those related to endogenous steroid production. For example, ACTH has been shown to be effective in the treatment of infantile spasms (IS), a condition in which corticosteroid treatment has limited effectiveness (Baram et al., 1996; Mackay et al., 2004; Stafstrom et al., 2011). Relevant to MS, emerging evidence indicates that ACTH exerts direct anti-inflammatory and immune-modulating effects within the central nervous system (CNS) and in peripheral tissues, and that these effects are mediated via the melanocortin receptors (Catania et al., 2004, 2010).

The objectives of this review are (1) to provide an overview of relapses, (2) to review the pathophysiology of MS with a focus on relapses, (3) to summarize current methods of relapse management, (4) to explore the actions of ACTH via the melanocortin system, and (5) to discuss the relevance of the melanocortin system to MS. These topics are addressed and discussed in the context of our collective clinical experience.

## Overview of Relapses

A relapse can be defined as an acute or insidious onset of new or worsening MS symptoms that are directly related to the disease and not attributed to environmental or systemic triggers, lasting for at least 24 h. Paroxysmal symptoms may be considered relapses if they consist of multiple episodes occurring over 24 h (Polman et al., 2011). Relapses vary in presentation and may include motor or sensory symptoms which may reflect the location of the demyelinating event and inflammation (Vollmer, 2007; Compston and Coles, 2008). The changes attributable to a relapse can persist for several weeks to months (Vollmer, 2007), although the time to recovery may be shortened with treatment (Filippini et al., 2000).

In clinical practice, the diagnosis and treatment of relapses may present many challenges. There are several types of relapses (e.g., optic neuritis, brainstem, myelitis), and even within each type of relapse, symptoms can vary (Frohman et al., 2008). Identifying relapses and ruling out pseudo-relapses in the presence of infection may be difficult (Vollmer, 2007; Thrower, 2009). Distinguishing between relapses and steady progression requires careful consideration, clinical skills, and judgment as the clinical manifestations of relapses as well as progressive symptoms can be quite similar (Frohman et al., 2008). Symptom presentation may provide clues for the clinician. For example, neuropathic pain such as tingling/pins-and-needle sensations may indicate a relapse (myelitis) if it is acute and/or transient, whereas chronic or insidious sensory changes or pain syndromes more likely reflect disease progression. Similarly, new onset sudden weakness of both legs affecting the ability to walk suggests a relapse whereas gradual change in walking ability over a long period of time suggests disease progression. Furthermore, treatment of relapses presents another challenge. Although short courses of high-dose corticosteroids are widely used and are generally accepted as the primary treatment option, for some patients there are limitations in terms of effectiveness, tolerability, and satisfaction with treatment outcomes (Nickerson and Marrie, 2011).

## Pathophysiology of MS

Multiple sclerosis is an immune-mediated disease leading to CNS inflammation, demyelination, and degeneration (Frohman et al., 2008; Boppana et al., 2011). Acute inflammatory demyelination and axonal loss in focal lesions are believed to be responsible for clinical manifestations of relapses (Noseworthy et al., 2000; Bruck, 2005a; Frohman et al., 2008). Chronic demyelination, axonal loss, and gliosis (i.e., a proliferation of astrocytes in damaged areas of the CNS, which usually leads to the formation of a glial scar) are associated, although not exclusively, with progressive MS, and are believed to contribute to chronic neurologic deficits (Bruck, 2005b; Frohman et al., 2005). Neurodegenerative effects associated with disease progression may be associated with inflammation and lesion formation (Frischer et al., 2009), however there may be processes that contribute to axonal loss separate from those that cause demyelination (Trapp et al., 1998; Bruck, 2005a; DeLuca et al., 2006). The following discussion focuses on acute demyelination and inflammatory processes relevant to relapses.

### Demyelination and inflammation

**Figure 1:**
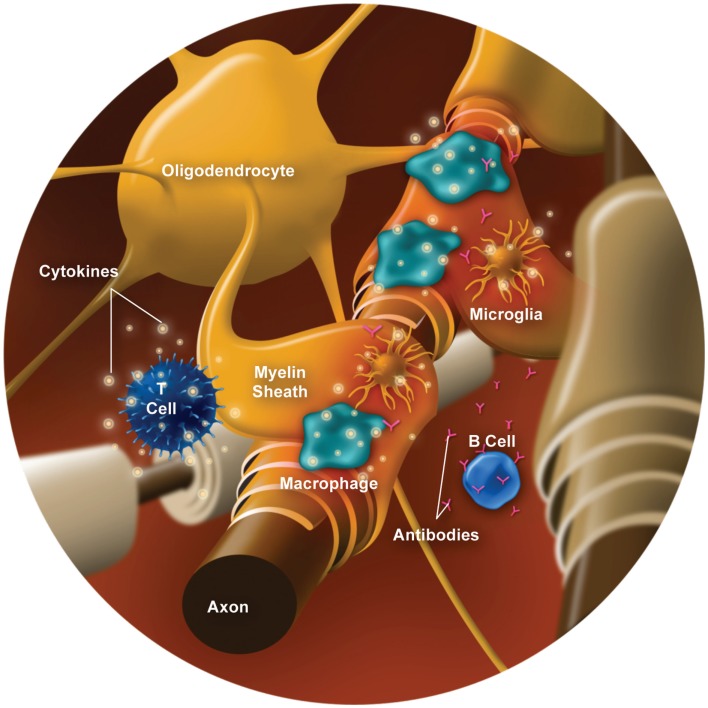
**Cells and inflammatory mediators involved in demyelination**.

Myelin, which is formed by oligodendrocytes in the CNS, surrounds and protects axons while functioning to facilitate signal conduction along the axon. Myelin damage leads to conduction slowing or completely blocked conduction, which underlies the clinical presentation of relapses. Demyelination involves several types of immune cells (e.g., T cells, B cells, and macrophages/monocytes) and inflammatory mediators (e.g., cytokines and chemokines; reviewed by Frohman et al., 2008; Boppana et al., 2011). Various contributors to CNS inflammation and demyelination are illustrated in Figure 1, and outlined below.

Increased blood-brain barrier (BBB) permeability is a hallmark of MS and is a key factor in the inflammatory process leading to demyelination and formation of MS lesions (Boppana et al., 2011). Immune and inflammatory cells that are activated in the periphery, possibly by myelin antigen(s) or microbial antigens cross-reactive with myelin, can increase BBB permeability, thereby allowing infiltration of proinflammatory cells (e.g., T cells and B cells) into the CNS. Penetration of immune cells into the CNS is promoted by enhanced expression of adhesion molecules and release of chemokines (e.g., CCL2, CCL5, CXCL1, CXCL2; Wilson et al., 2010) provide an informative summary) and substances like mixed metalloproteinases (MMPs) that promote the migration of immune cells and contribute to disruption of the BBB (Prat et al., 2002; Correale and Villa, 2007). From a clinical perspective, the appearance of gadolinium-enhancing MS lesions on magnetic resonance imaging is due to the permeability of the compromised integrity of the BBB; that is, gadolinium can only enter the CNS if the BBB is disrupted. The gadolinium “stains” the lesion in the CNS and presents it as an acute lesion that may correlate with the patients’ symptoms; however some Gd+ lesions are clinically silent unless they are large or involve eloquent areas of the CNS, e.g., an enhancing lesion in the brain stem.

T cells have a key role in MS (reviewed in Fletcher et al., 2010). CD4^+^ T helper cells (i.e., Th_1_, Th_2_, and Th_17_ cells) and CD8^+^ T cells have been shown to be present in MS lesions (Lassmann et al., 2007) and are implicated in the pathogenesis of MS (Mars et al., 2011). T cells in the CNS become activated (or re-activated) upon presentation of a myelin antigen, leading to secretion of cytokines and inflammatory mediators (Fletcher et al., 2010). Specifically, Th_1_ cells release the proinflammatory cytokines IFN-γ and tumor necrosis factor-α (TNF-α), which have several functions, including activation of macrophages (Boppana et al., 2011). Th_17_ cells produce interleukin (IL)-17A, IL-17F, IL-21, and IL-22, a key inflammatory mediator (Boppana et al., 2011). Regulatory T cells (T_reg_) normally control the intensity of the immune response and maintain self-tolerance through production of anti-inflammatory cytokines, such as IL-10 (Frohman et al., 2008; Awad and Stuve, 2010; Boppana et al., 2011). In addition, CD4^+^ Th_2_ cells produce IL-4, IL-5, and IL-13, that can inhibit macrophage function (Frohman et al., 2008; Awad and Stuve, 2010). There appears to be a dysregulation in levels or impairment of functioning of regulatory/anti-inflammatory T cells in MS (Costantino et al., 2008; Frohman et al., 2008; Correale and Villa, 2010; Mikulkova et al., 2011). T_reg_ reduction and dysfunction may be greater in RRMS compared with progressive MS (Venken et al., 2006). Further, this dysregulation may be particularly relevant to relapses, as suggested by evidence of decreased T_reg_ cells (Correale and Villa, 2010), as well as imbalances of proinflammatory and anti-inflammatory/regulatory cytokines during relapses (Hollifield et al., 2003).

B cells also appear to have a role in MS and demyelination (reviewed in Frohman et al., 2008; Berer et al., 2011; Boppana et al., 2011) as data have shown that B cells in the cerebrospinal fluid (CSF) correlate with early CNS inflammation in RRMS (Kuenz et al., 2008). B cells generate antibodies against myelin that activate the complement cascade, leading to opsonization (phagocytosis/destruction) of myelin and oligodendrocytes (Bruck, 2005a). Further, certain B cell subtypes (memory B cells) contribute to proinflammatory cytokine production (e.g., lymphotoxin and TNF-α; Duddy et al., 2007) and may act as antigen-presenting cells to activate T cell differentiation (Racke, 2008; Weber et al., 2010; Wootla et al., 2011). For example, a study of treatment naïve patients with RRMS demonstrated that memory B cells stimulated CD4^+^ T cell proliferation in response to neuro-antigens [myelin basic protein and myelin oligodendrocyte glycoprotein (MOG), a membrane protein expressed on oligodendrocytes; Harp et al., 2010]. Some B cells, however, can exert anti-inflammatory effects through production of regulatory cytokines (e.g., IL-10) and facilitating T_reg_ cell development (Duddy et al., 2007; Weber and Hemmer, 2010).

Macrophages and microglia are also implicated in the processes of inflammation and acute demyelination. Activated macrophages can damage myelin and oligodendrocytes through phagocytosis, which may be mediated via T cells or antibodies and complement (via B cells; Bruck, 2005a; Breij et al., 2008; Gandhi et al., 2010). Macrophages and activated microglia also release cytotoxic substances, such as nitric oxide (NO) and reactive oxygen species, which contribute to myelin damage (Glass et al., 2010) as well as cytokines and other inflammatory mediators (Hauser and Oksenberg, 2006; Gandhi et al., 2010). Macrophages and microglia can act as antigen-presenting cells, which present the antigen to naïve T lymphocytes and produce cytokines that stimulate differentiation of T cells into effector cells, which are responsible for executing immune functions (such as Th_1_, Th_2_, Th_17_, T_reg_, and others; Glass et al., 2010). Microglia can also present antigen to primed T cells, re-stimulating them to release inflammatory cytokines (Carson, 2002). In turn, activated T cells and cytokines, such as IFN-γ, can activate macrophages and microglia, which then release additional proinflammatory cytokines and chemokines (Sorensen et al., 1999; Carson, 2002; Boppana et al., 2011).

Remyelination following resolution of the acute inflammatory process is believed to contribute to recovery following a clinical relapse. However, remyelination is often incomplete (Bruck, 2005a), as oligodendrocytes are damaged by inflammatory processes (Chandran et al., 2008). Astrocytes proliferate and become hypertrophic in new lesions (Frohman et al., 2005) and form glial scars in areas where axonal transection has occurred (Frohman et al., 2008), which can be seen in chronic lesions (Frohman et al., 2006). Astroglial scarring impairs remyelination and repair (Carson et al., 2006; Frohman et al., 2006; Nair et al., 2008). Incomplete remyelination may contribute to the residual disability experienced by patients following resolution of a relapse; this disability may be primarily due to decreased conduction and damaged axons.

### Neurodegenerative processes

Neurodegenerative processes related to relapsing and progressive disease have not been fully elucidated. Although axonal damage has been demonstrated early in the course of MS, cumulative axonal damage over time appears to be an important contributor to clinical progression and sustained disability (Frohman et al., 2005; Petzold et al., 2005). Several potential mechanisms may be involved.

Axonal damage is seen early in MS lesion formation, suggesting that it may occur as a result of acute inflammatory demyelinating events (Bitsch et al., 2000; Bruck, 2005a). Multiple mediators of the immune response associated with acute inflammation and demyelination, including T cells, B cells, macrophages, cytokines, antibodies, NO, glutamate, and MMPs contribute to axon injury (Bruck, 2005a; Frohman et al., 2005). Accumulation of damage resulting from recurrent demyelinating events may account for some degree of axonal loss and disease progression.

Axonal injury may also develop independently of inflammatory demyelination (De Stefano et al., 2002). Although axonal injury/transection is most prominent in active demyelinating lesions (Trapp et al., 1998; Bruck, 2005b), acute axonal damage also occurs in partially remyelinating lesions and inactive demyelinated lesions (Bitsch et al., 2000). Cortical lesions (gray matter) show signs of neuronal loss (Hauser and Oksenberg, 2006), even though infiltration of inflammatory cells is lesser compared with white matter plaques (Bruck, 2005b). Further, axonal loss can occur without demyelination, as demonstrated by a study that showed injured axons with intact myelin (Bjartmar et al., 2001). Axonal damage in normal-appearing white matter may be due, in part, to Wallerian degeneration, which means damage distally to the injured plaques (Carson, 2002; Casanova et al., 2003; Bruck, 2005b; Lassmann, 2007). In patients with RRMS, the degree of axonal damage in normal-appearing white matter is significantly correlated with disability, suggesting that diffuse axonal damage outside of lesions may contribute substantially to chronic disability (Fu et al., 1998). Diffuse axonal injury in normal-appearing white matter is more pronounced in progressive MS compared with RRMS. This widespread injury is associated with a mild, but diffuse, inflammatory reaction, and extensive microglial activation (Kutzelnigg et al., 2005).

Finally, it has been suggested that chronically demyelinated axons may degenerate due to impaired regeneration potential or lack of trophic support from myelin and oligodendrocytes (Bruck, 2005b; Frohman et al., 2005). As noted previously, astroglial scar formation can impede regeneration (some evidence of axon regeneration has been observed in mouse models) and repair (Carson et al., 2006; Nair et al., 2008).

### The hypothalamic-pituitary-adrenal axis

**Figure 2:**
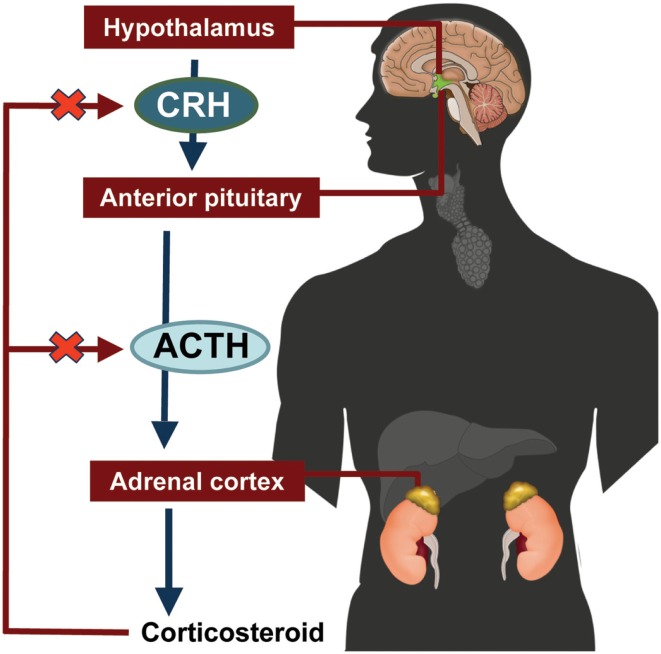
**Hypothalamic-pituitary-adrenal axis**. ACTH, adrenocorticotropic hormone; CRH, corticotropin-releasing hormone.

The hypothalamic-pituitary-adrenal (HPA) axis feedback loop is illustrated in Figure 2 (Jacobson, 2005; Papadimitriou and Priftis, 2009). In response to stress or injury, the hypothalamus releases corticotropin-releasing hormone (CRH), which stimulates the anterior pituitary gland to release ACTH. ACTH stimulates the adrenal cortex to secrete cortisol. Excess cortisol acts in a negative feedback loop to inhibit CRH and ACTH secretion by the hypothalamus and the anterior pituitary gland, respectively (Jacobson, 2005; Papadimitriou and Priftis, 2009).

Hypothalamic-pituitary-adrenal axis regulates many processes, including aspects of the immune response. The neuroendocrine and immune systems communicate bidirectionally (Chrousos, 1995; Haddad et al., 2002). Notably, cytokines, including several of those involved in the inflammatory processes previously described (e.g., TNF-α, IL-1), have a role in HPA axis regulation (Chrousos, 1995; Turnbull and Rivier, 1999; Haddad et al., 2002).

Some evidence shows that HPA axis function is dysregulated in patients with MS (Huitinga et al., 2003, 2004; Ysrraelit et al., 2008), although other data indicate normal HPA axis function (Limone et al., 2002). In addition to HPA axis hyperactivity (Ysrraelit et al., 2008) and chronic HPA activation (Huitinga et al., 2003), studies have also demonstrated impaired glucocorticoid receptor binding in lymphocytes (Then Bergh et al., 1999a; Ysrraelit et al., 2008), which may be a contributing factor to the suboptimal response in some patients to steroid treatment for relapses. Increased activity of HPA axis is also associated with fatigue (Gottschalk et al., 2005) and cognitive impairment (Heesen et al., 2002), which are commonly reported by patients experiencing relapses. The extent of HPA axis hyperactivity is associated with the clinical type of MS (Then Bergh et al., 1999b), and may be a predictor of progression (Gold et al., 2005a). Finally, neurodegeneration has been linked to HPA axis hyperactivity (Gold et al., 2005b; Gold and Heesen, 2006).

## Current Methods of Relapse Management

Approaches to relapse management vary widely, depending on number of factors such as the severity of relapse symptoms, patient or clinician preference, costs, and convenience. Some clinicians may choose not to actively treat relapses with mild or sensory symptoms that do not impair a patient’s daily functioning. For relapses that require treatment, options include high-dose corticosteroids, ACTH gel, plasmapheresis/plasma exchange (PLEX), and intravenous immunoglobulins (IVIG; Elovaara et al., 2008; National Clinical Advisory Board of the National Multiple Sclerosis Society, 2008; Cortese et al., 2011; H.P. Acthar^®^ Gel, 2011). High-dose corticosteroids are the standard of care for treating relapses; anti-inflammatory mechanisms and clinical use (dosing and side effects) are summarized below. ACTH gel may present an appropriate alternative to high-dose corticosteroid for some patients; its clinical use is summarized below and potential mechanisms of action (MOA) are explored later. PLEX is generally reserved for treatment of relapses that are severe or refractory to treatment with high-dose corticosteroids or ACTH, as evidence of its clinical efficacy is limited (Cortese et al., 2011). IVIG is sometimes used in patients for whom high-dose corticosteroids are contraindicated or not tolerated, but there is not consistent evidence for its use in relapse management (Elovaara et al., 2008). PLEX and IVIG are not discussed in detail.

As noted earlier, high-dose corticosteroids are the most common treatment for relapses. The efficacy of high-dose corticosteroids in speeding recovery from MS relapses has been demonstrated in several studies (Filippini et al., 2000). High-dose corticosteroids suppress the immunologic activation associated with MS relapses via several mechanisms (Sloka and Stefanelli, 2005). For example, methylprednisolone inhibits activation of T cells, decreases the influx of immune cells into the CNS, may facilitate apoptosis of activated immune cells, reduces production of inflammatory cytokines and indirectly decreases their cytotoxic effects, and reduces expression of several target genes related to inflammatory processes (Wandinger et al., 1998; Airla et al., 2004; Sloka and Stefanelli, 2005; Elovaara et al., 2006). Pulse therapy with intravenous methylprednisolone (IVMP) in patients with RRMS or progressive forms of MS may delay brain atrophy, slow progression of disability (Zivadinov et al., 2001), reduce T2 lesion volume (Then Bergh et al., 2006), and prevent confluence and enlargement of lesions (Zivadinov et al., 2008).

Common high-dose corticosteroid treatment regimens for relapses include IVMP (1 g/day for 3–5 days) or oral prednisone (1000–1250 mg/day for 5 days; National Clinical Advisory Board of the National Multiple Sclerosis Society, 2008; Morrow et al., 2009). Oral administration may offer greater convenience and ease of use for patients. Adverse events associated with high-dose corticosteroids include infection, hyperglycemia, mood effects (euphoria, psychosis), gastrointestinal symptoms, taste disturbances, insomnia, weight gain, edema, and hypertension; potential long-term effects include osteoporosis, cataracts, glaucoma, Cushingoid features, immune suppression, hypernatremia, and hypokalemia; rarely, patients treated with corticosteroids may develop avascular necrosis (AVN) of major joints, even after one dose of corticosteroid (Lyons et al., 1988; National Clinical Advisory Board of the National Multiple Sclerosis Society, 2008).

Adrenocorticotropic hormone gel, as previously noted, may be considered as an alternative treatment of MS relapses. In our experience, reasons for using ACTH include a lack of benefit established with corticosteroids, inability to tolerate corticosteroids or adverse effects, and poor IV access. Its efficacy has been studied in several published investigations, and treatment outcomes may be comparable to those seen with corticosteroids (Filippini et al., 2000).

Adrenocorticotropic hormone gel is usually given at a dose of 80 U daily for 5 days, administered subcutaneously (SQ) or intramuscularly (IM). The option of IM or SQ can be adjusted to patients’ experience with injections (e.g., if a patient is on IM interferon, ACTH gel can be prescribed as IM and if a patient is on glatiramer acetate, ACTH gel can be prescribed as SQ), to increase its ease of use for patients. The side effect profile of ACTH is generally similar to that of corticosteroids (Filippini et al., 2000), and includes fluid retention, alteration in glucose tolerance, elevation in blood pressure, behavioral and mood changes, increased appetite, and weight gain (see also list above for potential effects related to steroidogenesis; H.P. Acthar^®^ Gel, 2011); ACTH may have milder effects on bone and less risk of AVN (Zaidi et al., 2010). Before discovery of melanocortin receptors, ACTH gel was believed to work entirely by stimulating endogenous steroid production (National Clinical Advisory Board of the National Multiple Sclerosis Society, 2008; Sibley, 2009). However, existing and emerging evidence related to this system indicates additional effects of ACTH that may be relevant in terms of the MOA of ACTH gel for treatment of relapses.

## Overview of Melanocortin System

Melanocortins (MC) and their receptors (MCR) compose an ancient regulatory system, in existence for 500 million years (Heinig et al., 1995; Klovins et al., 2004; Takahashi and Kawauchi, 2006; Haitina et al., 2007; Catania, 2008), that exerts multiple influences on the host, including anti-inflammatory, immunomodulatory, and behavioral effects (Catania et al., 2004).

**Figure 3:**
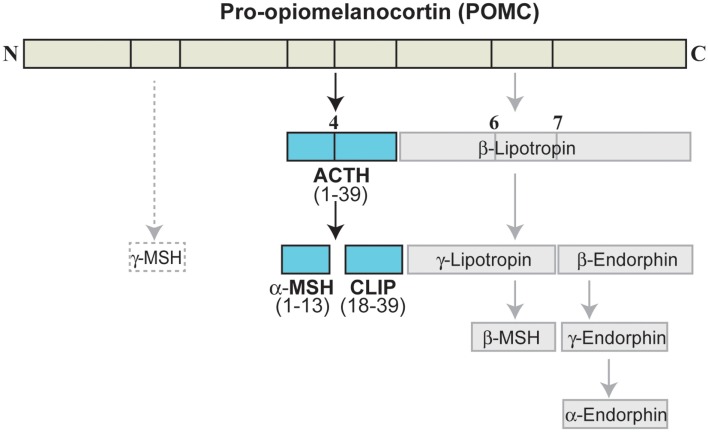
**Melanocortin peptides**. ACTH, adrenocorticotropic hormone; CLIP, corticotropin-like intermediate lobe peptide; MSH, melanocyte-stimulating hormone.

The MC are a peptide family that includes ACTH (or corticotropin) and the melanotropins α-, β-, γ-melanocyte-stimulating hormone (α-, β-, γ-MSH). The MC are derived from the precursor POMC, which is synthesized in the pituitary, the arcuate nucleus of the hypothalamus, the solitary tract of the medulla, and several peripheral tissues (Papadimitriou and Priftis, 2009). Cleavage of POMC by prohormone convertases 1 and 2 (PC1 and PC2) in the anterior pituitary leads to formation of ACTH, which can be further cleaved to generate α-MSH (Figure 3; Adan and Gispen, 1997; Papadimitriou and Priftis, 2009). Full sequence ACTH comprises 39 amino acids [i.e., ACTH_(1–39)_], of which α-MSH_(1–13)_ shares the 1–13 amino acid sequence; all of the MC share an invariant sequence of four amino acids (His-Phe-Arg-Trp), which contributes to some overlap in terms of receptor recognition and function (Catania et al., 2004).

There are five MCR subtypes (MC1R–MC5R), that are expressed in various tissues throughout the body (Gantz et al., 1993; Fathi et al., 1995; Xia et al., 1995; Chhajlani, 1996; Suzuki et al., 1996; Chen et al., 1997; Taherzadeh et al., 1999; Lindskog et al., 2010; Zaidi et al., 2010). The MCRs differ in their affinity for individual MC peptides (Catania et al., 2004; Brzoska et al., 2008). Notably, ACTH is the only MC peptide that is recognized by all MCRs and it is the only MC peptide recognized by the MC2R receptor in the adrenal cortex. The steroidogenic effects of ACTH are mediated via activation of these receptors (Catania et al., 2004). MCR affinity, distribution, and principal functions are summarized in Table 1. This discussion focuses on the effects of ACTH on the MC system that are potentially relevant to its therapeutic MOA; however, it should be noted that due to the wide distribution of MCRs and broad effects on multiple systems, ACTH may have additional actions that are relevant in terms of potential for side effects. For a comprehensive review of MC, see Catania et al. (2004) or Brzoska et al. (2008).

**Table 1:** **MCR distribution, affinity, and functions**.

MCR	Ligand affinity	Prevalent tissue	Functions
MC1R	α-MSH = ACTH >> γMSH	Melanocytes Immune/inflammatory cells Keratinocytes Endothelial cells Glial cells (CNS)	Pigmentary effects; antipyretic/anti-inflammatory
MC2R	ACTH	Adrenal cortex Osteoblasts	Steroidogenesis; bone formation(?)
MC3R	γ-MSH = ACTH ≥ α-MSH	CNS Macrophages	Autonomic functions; anti-inflammatory
MC4R	α-MSH = ACTH >> γMSH	CNS	Control of feeding/energy; neuroprotection; Erectile activity
MC5R	α-MSH ≥ ACTH > γMSH	Exocrine glands Lymphocytes (T and B cells)	Regulation of exocrine secretions; immunoregulatory functions

*ACTH, adrenocorticotropic hormone; CNS, central nervous system; MCR, melanocortin receptor; MSH, melanocyte-stimulating hormone*.

*Adapted from Catania et al. (2004) reprinted with permission*.

Of particular relevance to MS, several types of immune cells express MCRs. For example, peripheral T helper cells and monocytes express MC1R, MC2R, MC3R, and MC5R mRNA (Andersen et al., 2005); MC1R, MC2R, and MC3R have also been shown on B cells (Johnson et al., 2001; Cooper et al., 2005); and MC2R on macrophages (Johnson et al., 2001).

## Functions of Melanocortin System Relevant to MS

Various anti-inflammatory/immunomodulatory functions are mediated via the MC system (Luger and Brzoska, 2007), some of which may be relevant to the pathophysiologic processes involved in MS. These effects are seen with various cell types (e.g., T cells and macrophages) and other pathways (e.g., sympathetic nervous system signaling) involved in inflammatory processes (or regulation of inflammatory processes) implicated in MS. For example, MC peptides have been shown to act directly to reduce production of cytotoxic molecules and inflammatory mediators in activated microglia (murine cells Delgado et al., 1998); and macrophages (human Taherzadeh et al., 1999); and to enhance immunoregulatory functions of T cells (Taylor and Kitaichi, 2008). MC peptides also exert indirect effects via potentiation of sympathetic/adrenergic pathways (Gothert, 1984; Szabo et al., 1989; Macaluso et al., 1994; Nankova et al., 1996; Ichiyama et al., 1999a; Serova et al., 2008) and modulation of neurotransmitters (Pranzatelli, 1994). These and other effects are described in more detail below.

It should be noted that much of the evidence for melanocortin effects on immune function comes from *in vitro* studies and studies of animal models or conditions other than MS. However, these functions comprise biologically conserved sequences and pathways that are consistent across multiple species and cell types; thus it is reasonable to expect that these results may extrapolate to many types of inflammatory processes, including those implicated in MS. Similarly, most evidence is based on studies that used α-MSH; this melanocortin peptide is often selected for use in studies because it does not stimulate MC2R and therefore its effects are not related to stimulation of corticosteroid production. However, given the overlap in amino acid sequences and MCR affinity, it would be expected that ACTH would have similar effects. Finally, although evidence demonstrates the efficacy of ACTH in speeding recovery from MS relapses, the specific physiological changes induced by modulation of the MC system activity in patients with MS have not yet been studied.

### Impact on cells and processes involved in MS

Adrenocorticotropic hormone and other melanocortin peptides reduce production of proinflammatory cytokines and chemokines (e.g., IL-1, IL-8, TNF-α) and other mediators of inflammatory processes (e.g., NO, adhesion molecules; Mason and Van, 1989; Chiao et al., 1996; Catania et al., 1998; Delgado et al., 1998; Böhm et al., 1999, 2005; Taherzadeh et al., 1999; Scholzen et al., 2003; Manna et al., 2006). For example, in a study using a murine cultured cell line, α-MSH_(1–13)_, α-MSH_(11–13)_, and ACTH_(1–24)_ inhibited production of TNF-α, IL-6, and NO by activated microglia (Delgado et al., 1998). Inhibition of TNF-α production by α-MSH_(1–13)_, ACTH_(1–24)_, and ACTH_(1–39)_ was also demonstrated in human monocytes and macrophages (Taherzadeh et al., 1999). In human neutrophils and macrophages, α-MSH inhibited several IL-8-mediated functions, including chemotaxis, enzyme release, and generation of reactive oxygen (Manna et al., 2006). This is a momentous effect as IL-8 is a key mediator of inflammation and is also involved in the development and progression of cancer (Waugh and Wilson, 2008). ACTH and MC may also promote the anti-inflammatory processes. For example, α-MSH increases expression of the anti-inflammatory cytokine IL-10 mRNA in keratinocytes and induces production of IL-10 in human monocytes (Bhardwaj et al., 1996; Redondo et al., 1998). Thus, in MS, ACTH may act by modulating macrophage and other cell activity and cytokine levels; the consequent reduction of central inflammation may protect myelin and axons from destruction.

A key mechanism underlying the anti-inflammatory effects of MC peptides appears to be the modulation of nuclear factor (NF)-κB signaling (Manna and Aggarwal, 1998; Ichiyama et al., 1999b; Scholzen et al., 2003). NF-κB is a transcription factor that regulates expression of several genes involved in immune and inflammatory processes (e.g., those that code for proinflammatory cytokines, MMPs, adhesion molecules, and inflammatory enzymes), NF-κB is retained in an inactive form in the cytoplasm, bound to members of the IκB inhibitory protein family. Phosphorylation of IκB by various agents such as cytokines, bacterial products, and viruses causes IκB degradation. Subsequently, the free NF-κB is translocated to the nucleus where it binds to sequences of DNA encoding NF-κB-responsive elements and triggers the transcription of target genes (Beg et al., 1993; Baldwin, 1996). Because NF-κB is found in many cell types relevant to MS, inhibition of this factor could result in regulation of inflammation via several avenues (Yan and Greer, 2008). In lymphocytes stimulated with lipopolysaccharide (LPS), α-MSH reduced NF-κB activation, adhesion molecule expression, and adhesion molecule-mediated adhesion of lymphocytes (Scholzen et al., 2003). LPS-induced TNF-α production in leukocytes was likewise inhibited by α-MSH (Yoon et al., 2003). NF-κB inhibition is achieved via MC modulation of cytokine activity, as shown in a study demonstrating that α-MSH inhibited IL-8-induced NF-κB activation in human macrophages (Manna et al., 2006). In addition, parenteral administration of α-MSH inhibited NF-κB activation in a murine model of experimental acute brain inflammation (Ichiyama et al., 1999b). This finding is also relevant for MS treatment as it suggests that MCs such as ACTH administered peripherally, can exert significant anti-inflammatory effects within the brain.

Adrenocorticotropic hormone and other MCs exert immunomodulatory effects by affecting T cell functions. For example, in studies on experimental autoimmune encephalomyelitis (EAE, a mouse model for MS), orally administered ACTH induced expression of T_reg_ cells, increased secretion of immunoregulatory IL-4, and decreased IL-17, IL-2, and IFN-γ in CNS lymphocytes (Brod and Hood, 2011), while α-MSH induced T cells to produce regulatory cytokines (Taylor and Kitaichi, 2008). In addition, studies of experimental autoimmune uveitis have shown that α-MSH mediates the induction of T_reg_ cells and modulates T cell phenotype, essentially converting primed T cells to act as T_reg_ cells (Taylor and Namba, 2001; Namba et al., 2002). This effect appears to be mediated via the MC5R on the primed T cells (Taylor and Namba, 2001). In MS, decreasing the signaling of T helper cells may reduce recognition of the self-antigen; likewise, increasing signaling of T_reg_ cells may help to control the number of autoimmune antigen-presenting cells.

### Effects on sympathetic nervous system and neurotransmitters

The role of the sympathetic nervous system in immune responses and autoimmune diseases is demonstrated by the exacerbation of EAE following chemical ablation of the sympathetic nervous system (Chelmicka-Schorr et al., 1988). Immune cells, including lymphocytes, macrophages, and microglia, express β_2_-adrenergic receptors (Schorr and Arnason, 1999), the activation of which leads to signals that help to control inflammation. This is illustrated by studies showing that β_2_-adrenergic agonists inhibit macrophage functions, including reduced production of TNF-α (Chelmicka-Schorr et al., 1988; Schorr and Arnason, 1999). The observation that β_2_-adrenergic agonists suppress EAE (Wiegmann et al., 1995) suggests that the sympathetic nervous system effects may be relevant to MS. Although β_2_-adrenergic agonists are not routinely used in MS treatment, one study found that add-on treatment with albuterol in MS patients receiving glatiramer acetate was associated with improvement in clinical outcomes, including relapse rates, and MS Functional Composite (MSFC) scores at 6 and 12 months (Khoury et al., 2010).

Adrenocorticotropic hormone and other MC can activate anti-inflammatory signals via potentiation of sympathetic/adrenergic pathways (Gothert, 1984; Szabo et al., 1989; Macaluso et al., 1994; Nankova et al., 1996; Ichiyama et al., 1999a; Serova et al., 2008). For example, ACTH has been shown to increase expression of tyrosine hydroxylase [an enzyme involved in norepinephrine (NE) synthesis] mRNA in rat brain (Nankova et al., 1996; Serova et al., 2008). Additionally, centrally administered α-MSH potentiates peripheral beta-adrenergic anti-inflammatory effects via descending neural pathways (Macaluso et al., 1994; Ichiyama et al., 1999a). Further, MC peptides stimulate increased release of NE from peripheral sympathetic nerve endings (Gothert, 1984; Szabo et al., 1989).

In addition to increasing synthesis of NE in the CNS (Nankova et al., 1996; Serova et al., 2008), ACTH modulates synthesis, release, and actions of other neurotransmitters, including dopamine and acetylcholine, within the CNS (Pranzatelli, 1994). Receptors for several of these neurotransmitters have been identified on microglia and macrophages; engagement of these receptors has inhibitory effects on microglia (Chang and Liu, 2000; Haskó et al., 2002; Shytle et al., 2004; Färber et al., 2005). Potentiation of neurotransmitter signaling in the brain by ACTH or other MC may stimulate these inhibitory receptors, thereby reducing microglial activation. As noted previously, microglia are an important component of the inflammatory mechanisms leading to demyelination and lesion formation in MS; (Carson, 2002; Boppana et al., 2011) thus, this may be one mechanism by which ACTH works in treating relapses. In addition, widespread microglial activation has been demonstrated in secondary progressive MS (SPMS; Kutzelnigg et al., 2005), suggesting that ACTH may be of benefit in the progressive phase of MS; however, this has not been studied in clinical trials.

One issue in considering the evidence described here in terms of its applicability to clinical practice, is that many of the studies were performed *in vitro* or in animal models in which ACTH or α-MSH was administered directly into the brain. This is in contrast to the clinical situation in which ACTH is administered peripherally (IM or SQ), which may raise the question of whether ACTH gel can actually have effects on the CNS. However, the permeability of the BBB is compromised in MS, particularly during relapses (Kermode et al., 1990; Prat et al., 2002), which would allow peripherally administered ACTH to pass through. Further, because MC4Rs are expressed throughout the CNS and on vagal sensory afferent nerves that project to the CNS (Gautron et al., 2010), ACTH in the periphery can have central effects. In fact, there is evidence demonstrating that systemic administration of ACTH affects behavior and neurotransmitters (Sandman and Kastin, 1981; de Wied and Jolles, 1982; Serova et al., 2008). Finally, from a clinical perspective, the efficacy of ACTH gel in other conditions, such as IS, demonstrates its ability to affect the brain, as it is a disease confined to the brain (Stafstrom et al., 2011).

Thus, the clinical experience of effectiveness of ACTH gel in cases of corticosteroid non-responsive patients treated for relapse may be explained, at least in part, by the actions of ACTH via the MC system.

## Emerging Evidence and Future Directions

Although it is beyond the scope of this review, additional research endeavors have begun to examine other aspects of ACTH/MC signaling that may be relevant to MS. For example, some evidence suggests that vagal nerve involvement may play a role in anti-inflammatory/immunomodulatory effects (Richman and Arnason, 1979; Adan and Gispen, 1997; Kishi et al., 2003; Guarini et al., 2004; Pavlov et al., 2006; Wan et al., 2008; Gautron et al., 2010). Evidence suggesting neuroprotective effects of ACTH/MCs (Strand and Kung, 1980; van der Neut et al., 1992; Catania, 2008), raises the possibility for a role of ACTH gel in treatment of progressive forms of MS. Bone maintenance functions mediated via the MC system (Zaidi et al., 2010) may be relevant in light of bone loss related to immobility associated with MS, as well as the potential for bone loss related to side effects of corticosteroid or ACTH treatment.

Future directions for additional research may include effects of ACTH and other MC on cognition, as cognitive dysfunction is an important aspect of MS. Investigations of MC functions related to strength, mobility, and other functions would be of interest to the MS community.

## Conflict of Interest Statement

Amy Perrin Ross has received a consulting fee or honorarium and support to travel to meetings from Questcor and is a consultant for Acorda, Allergan, Teva, Questcor, EMD Serono, and Genzyme. Aliza Ben-Zacharia has received a consulting fee or honorarium and support to travel to meetings from Questcor and is a consultant for Acorda, Teva, Questcor, EMD Serono, Biogen, and Genzyme. Colleen Harris is a consultant for and has received a consulting fee or honorarium from Questcor. Jennifer Smrtka is a consultant for CANDOMS Organization, PRIME Organization, CMSC Organizational, and Consensus Medical Corporation; has received payment for lectures including service on speakers bureaus for Acorda, Bayer, EMD Serono, Novartis, Pfizer, Genzyme, Questcor, and Teva; and has received payment for development of education presentations from PRIME.
